# Glycemic index of some local staples in Ghana

**DOI:** 10.1002/fsn3.372

**Published:** 2016-04-18

**Authors:** Divine Eli‐Cophie, Jacob K. Agbenorhevi, Reginald A. Annan

**Affiliations:** ^1^ Department of Biochemistry and BiotechnologyKwame Nkrumah University of Science and TechnologyKumasiGhana; ^2^ Department of Food Science and TechnologyKwame Nkrumah University of Science and TechnologyKumasiGhana

**Keywords:** *Banku*, Carbohydrate foods, *Fufu*, Glycemic index, *Kenkey*, *Tuo Zaafi*

## Abstract

Glycemic index (GI), a measure of blood glucose level as influenced by foods has become a concern due to the increasing cases of diabetes in Ghana. In spite of this, little is known of the GI of commonly consumed carbohydrate‐rich foods of the Ghanaian diet. The GI of five Ghanaian staples: *fufu* (locally pounded), *kenkey* (Ga), *banku*,* Tuo Zaafi* (TZ), and *fufu* (Processed powder) were determined in a crossover trial among 10 healthy nondiabetics. Participants were given 50 g portions of pure glucose on two different occasions and subsequently the test foods containing 50 g available carbohydrates. Capillary blood glucose levels of the subjects at fasting and after ingestion of the glucose and test foods were measured within a 2‐hour period. The GI of the test foods were calculated by dividing the incremental area under the glucose response curve (IAUC) of the test food by the IAUC for the reference food and multiplying the result by 100. Processed‐powdered *fufu* had the least glycemic response (31), followed by Ga *kenkey* (41) and locally pounded *fufu* (55), all recording low GI. *Tuo Zaafi (68)* had a medium GI and *banku (73)*, moderately high GI. Comparison of GI between the foods using ANOVA revealed a significant difference between GIs of locally pounded *fufu* versus I‐*fufu* (industrially processed *fufu* flour) (*p* = 0.026). This study showed that the five major Ghanaian staples showed low to moderately high GI. These should be considered in recommendations for diabetics.

## Introduction

Carbohydrates which are the main energy source in most human diets, making up about 40–80% of our calorie intake play an enormous role in human physiology (Mann et al. 2007). Most Ghanaian diets are carbohydrate based and most families plan their meals around it. Despite the energy value of carbohydrates, its physiological effects on human health cannot be overemphasized. The energy contents and digestibility of different carbohydrates, however, differ (Mann et al. 2007). Some carbohydrate foods elicit a quicker response from insulin than others (Lin et al. 2010). This is due to differences in the rate at which they release glucose into the blood. The relative ranking of how fast or slow a carbohydrate food is converted to glucose after ingestion is a measure of its glycemic index (Lavigne et al. 2000). Glycemic index (GI), though a simple numerical index which measures the blood glucose raising ability of carbohydrates, has become an established concept for classifying carbohydrates (FAO/WHO, 1998). In determining the glycemic index of a carbohydrate food, the postprandial glycemic response of the food is measured against a reference food (FAO/WHO, 1998). A number of factors influence the postprandial glycemic response of a carbohydrate when ingested. These factors range from extrinsic components such as composition of the whole meal and variations in the overall diet, to intrinsic properties, such as the amylose to amylopectin ratio, presence or absence of viscous fiber, and the length of the monosaccharide units (Bjorck et al. 1994).

Most Ghanaian carbohydrates (such as corn, rice, cassava, yam, and plantain) are subjected to quite a number of processing techniques during preparation for consumption. The processing of a particular carbohydrate food plays an important role in determining its overall properties (Englyst et al. 2007), which also has a significant influence on physiological function in the human body. Glycemic index value is also directly influenced when the physiological effect of a carbohydrate is altered (Bahado‐Singh et al. 2011). Such factors as particle size, processing methods, nature of starch, and antinutrients present which are not commonly available in food tables, and yet have very significant effects on physiological properties of food, which further highlight the importance of determining the GI of foods individually and not by extrapolation from international GI values of foods of similar qualities (Aston et al. 2008).

The study aim was to determine the GI of some carbohydrate‐rich Ghanaian staples.

## Methodology

The research was approved by the Committee on Human Research, Publication and Ethics of the Kwame Nkrumah University of Science and Technology School of Medical Sciences/Komfo Anokye Teaching Hospital. Ten (10) apparently healthy, nondiabetic human subjects (eight males and two females) between 20 and 50 years were recruited for the clinical trial upon obtaining their informed consent. The method of measurement and calculation of GI was in line with WHO/FAO recommendations for determination of Glycemic Index (FAO/WHO, 1998). The subjects had no known diseases nor were on any medications which could influence the results of the study. Participants were informed of a strict abstinence from smoking or drinking within the period of the study. All subjects fasted 10–14 h from the previous night to the morning of testing and did not engage in strenuous physical activity prior to testing days. Last meal and time eaten, by subjects the previous night were documented.

On reporting for the first test appointment, height, weight, and waist circumference of each respondent were taken. Afterward, a rounded drop of capillary blood was taken from each participant by a prick of the finger, to assay for fasting blood sugar (FBS) using an Ultra 2 glucometer. All the subjects were given 50 g glucose solution with 200 mL of water. Blood glucose levels were measured similarly at 15, 30, 45, 60, 90, and 120 min after consumption of glucose solution. Respondents reported every other day for testing of the test foods. On reporting, FBS of each respondent were measured, after which they were given measured amounts of test foods containing 50 g available carbohydrate portion (calculation of available carbohydrate portions were based on proximate analysis done by Eyeson et al. 1975). All test foods were eaten with about 110 g of the same light soup and a serving of salmon fish which provided approximately 2 g of available carbohydrate portions. The reference food, glucose was administered a second time to the subjects on the fourth testing session. In all, five foods: *fufu* (locally pounded), *kenkey* (Ga), *banku*,* Tuo Zaafi* (TZ), *fufu* (Processed powder), prepared following standard indigenous preparation methods were tested under the same preconditions and procedure.

Locally pounded *fufu* (LPF) preparation involved plantain and cassava quantities in the ratio of 80:20 boiled and pounded into paste. *Banku* was prepared from corn dough and cassava dough in the ratio of 80:20 stirred in hot water to form a palp. *TZ* and *kenkey* were, however, solely from corn. *TZ* is made from unfermented maize flour stirred in hot water into palp. *kenkey* is made from fermented corn dough. Industry‐processed *fufu* flour, containing the following ingredients: plantain, cassava, and potato was, however, prepared based on instruction on the package.

### Data analysis

The incremental area under the glucose response curves (IAUC) were calculated using the trapezoid rule as recommended by FAO/WHO (FAO/WHO, 1998). The area under the fasting baseline was ignored in the calculation. All GIs that were 2SD above or below the mean GI value for a given test were ignored as outliers (Wolever et al. 2011). The IAUC for each test food was expressed as a percentage of the mean IAUC of the single repeat of glucose which was the reference food used. The GI of each test food was calculated as the mean GI as obtained by each subject in the study that consumed the test food. Glycemic Index Classes: Foods were classified as low, medium, or high GI according to the following: GI values ≤55; Low GI, 56–69; Medium and GI, ≥70; High GI (Bahado‐Singh et al. 2011). The glucose response curves were plotted with the GraphPad Prism software version 5.00 (GraphPad Prism Inc., San Diego, California, US). Data were analyzed using Microsoft Excel and Statistical Package for Social Sciences (SPSS) software version 20 (IBM SPSS Statistics Inc., North Castle, New York, US).

## Results

The mean age of the respondents was 30.9 ± 6.4 years (range: 24–46 years), mean body mass index (BMI) of 26.96 ± 5.2 kg/m^2^ (range: 22.2–39.1), and mean waist circumference (WC) was 88.6 ± 13.84 cm (range: 77–122).

**Figure 1:**
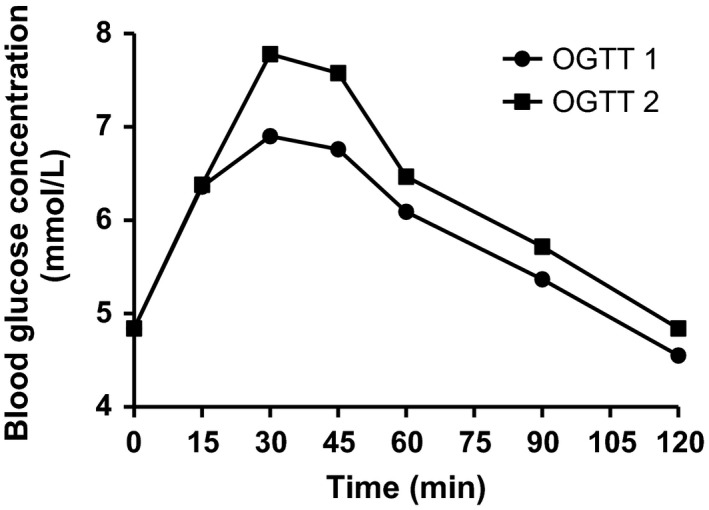
Mean glycemic response elicited by 50 g glucose in duplicate.

**Figure 2:**
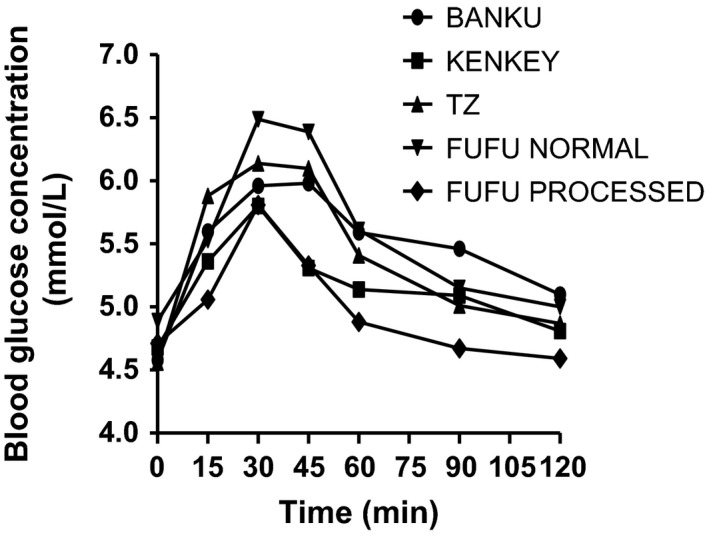
Mean glycemic responses elicited by study subjects after consumption of 50 g available carbohydrates portions of all test foods.

Figure 1 and 2 illustrate the blood glucose response elicited by the reference food (50 g glucose) and the test foods, respectively. Table 1 shows the mean IAUC of glucose and the test foods for each participant. Glucose recorded the highest mean IAUC of 158.8. Of the tested foods, industrially processed *fufu* (IPF) had the lowest IAUC of 47.9, whereas *banku* recorded the highest of 115.7.

**Table 1:** Incremental area under the curve of the test and reference foods by the study subjects

Subject	Incremental area under the glucose response curve (IAUC)					
	Glucose mean	*Banku*	*Kenkey*	*Tuo Zaafi (TZ)*	Pounded *fufu*	Processed *fufu*
VK01	156.08	125.25	63.75	154.5	124.5	105
JK03	112.13	74.14	26.59	54	32.4	11.36
IA04	92.57	58.61	49.2	84.75	92.25	16.05
EL05	112.88	–	–	–	–	48.75
AD06	292.88	152.25	18.94	119.25	104.25	43.75
JA07	104.4	102.75	46.5	60.19	64.5	26.25
CP08	152.38	92.25	44.63	66.75	56.67	77.25
PB09	206.45	180.75	153.75	195	82.38	83.25
BA10	131.49	109.5	46.5	84		20.25
PA11	226.87	145.5	136.5	157.15	123.75	–
MEAN	158.8 ± 64.4	115.7 ± 39.2^X^	65.2 ± 47.4^XY^	108.4 ± 47.5^X^	85.1 ± 32.7^XY^	47.9 ± 33.5^Y^

^XY^Values in the same row having no common superscript differ significantly (*P *<* *0.05).

The GI of all the foods tested, their standard error, and GI group are represented in Table 2. Glycemic index of the foods ranged from low (≤55) through medium (56–69) to high (≥70). The three corn‐based foods, *banku*,* TZ*, and *Kenkey* had high GI, medium, and low GI, respectively. There were no significant differences between the GI of *banku* and *TZ* or *banku* and locally pounded *fufu* (LPF) (*P > *0.05); however, the GI of *banku* differed significantly from *kenkey* (*P < *0.05) which is also corn based. There was a significant difference between the GI of LPF and *fufu* made from industry‐processed *fufu* flour (*p = 0.026*), both of which are processed quite differently. The GI value of *fufu* prepared from industry‐processed flour was the least (GI* = *17–48 at 95% CI) (Table 2). Although both industry‐processed *fufu* and locally pounded *fufu* had low GI, the IAUC of locally pounded *fufu* was about 33%, significantly higher than the IAUC of industry‐processed *fufu* (Table 1).

**Table 2:** Glycemic Index and Class of Selected Staples

No.	Food item	At 95% CI		GI (%)	SE	GI class
		GI_min_ (%)	GI_max_ (%)			
1	Glucose	100	100	100	0.0	H
2	*Banku*	61	85	73^A^	5.0	H
3	Kenkey	25	55	41^B,C^	6.8	L
4	*Tuo Zaafi (TZ)*	50	84	68^A,C^	7.5	M
5	Pounded *fufu*	30	76	55^A,C^	8.7	L
6	Processed *fufu*	17	48	31^B^	6.6	L

^A,B,C^Values in the same column with no common superscript differ significantly (*P* < 0.05). GI Class (Group): Low (L); Medium (M); High (H); SE, standard error.

The average peak of postprandial glucose in all subjects after consumption of test food was observed at the 30th min from ingestion of food as was observed with the pure glucose solution.

From Figure 1 and 2, there were not much difference in the fasting blood sugar levels before any of the test or reference foods were consumed, though the subjects consumed different meals the evenings prior to testing days. A critical assessment of the previous evening meals of subjects in this study revealed one subject (IA 04) who had taken a high fiber diet, oats, the evening prior to testing of *banku*. Though subject IA 04 did not exclusively have the least glucose response for all the foods tested, the IAUC of *banku* for that subject was observed to be the least compared to the other subjects (Table 1).

We assessed the possible factors (intrinsic and extrinsic) that might have influenced the GI of the various foods.

### Effect of portion size

Although the portion sizes of the test foods differed, they were all calculated to provide 50 g available carbohydrates (Lin et al. 2010). The GI of *banku* which had the largest portion size (287 g) per 50 g available carbohydrate was the highest among the foods. *Kenkey* on the other hand had a larger 50 g available carbohydrate portion size (189 g) than *fufu* (153 g) but elicited a lower *glycemic* response than *fufu* (LPF). Also, the amount of LPF and IPF ingested by subjects were the same (153 g), but the AUC of the LPF was about 33% higher than that of IPF (Table 1). This affirms the position that when the foods contain similar amounts of available carbohydrate, then the portion size plays a less significant role.

### Effect of fiber

The fiber content of freshly harvested 100 g cassava, plantain, and maize are 1.8 g, 2.3 g, and 7.3 g, respectively (United States Department of Agriculture, 2002), all of which are higher than in their processed form as *banku*,* TZ*,* kenkey,* or *fufu*. Comparing the fiber contents, weight for weight, there is a noticeable difference in the fiber content before and after processing into various dishes. The fiber content of *banku*,* TZ,* and *kenkey* were 0.1, 0.3, and 1.3 g per 100 g and their GIs were 73, 68, and 41, respectively (Englyst et al. 2007). The GI of *kenkey* which had the highest fiber content was significantly lower than that of *banku* (*P *=* *0.024). The fiber content of *fufu* was 0.1 g as with *banku*, but had a lower glycemic response than *banku* of similar fiber content and *TZ* of comparatively higher fiber content (Table 2).

## Discussion

Most staple foods of the various regions in Ghana are carbohydrate rich. Classification of foods based on their respective glycemic responses has helped to clear the erroneous perception that carbohydrate‐rich foods are the bane of most persons with metabolic disorders. The increasing number of evidence‐based research affirms the assertion that not all carbohydrates are of the same quality (Bahado‐Singh et al. 2011). The foods tested: *banku*, Ga *kenkey*,* Tuo Zaafi*, and *fufu* are the main staple foods consumed by the people of the Volta, Greater Accra, Northern, and Ashanti Regions, respectively. However, these foods are now widely consumed throughout the country and may be considered the five major carbohydrate‐rich staple foods consumed in Ghana. Thus, this study fills a major gap of knowing the GI of these major carbohydrate‐rich foods.

Of all the foods tested, *banku* had the highest GI value (73), followed by *TZ* (68), and locally prepared *fufu* (55), *kenkey* (41), and the least being *fufu* (31) prepared from industry‐processed *fufu* flour (Table 2). All these foods were prepared by going through boiling of one form or the other. This implies that the GI and therefore carbohydrate quality of Ghanaian staples vary and this should inform choice in managing blood sugar.

During the study, participants complained of a nauseating feeling after consumption of glucose solution which was the reference food. Similar response from participants was reported by Brouns et al. (2005), who studied the use of white bread and glucose as reference foods in GI determination. However, glucose is still adjudged as the best reference food because of potential variations that could result in the preparation of white bread in different research areas (Bornet et al. 1987).

This study has established that the processing methods influence the GI of the foods. This is because, even though *kenkey* and *banku* are both maize based, *kenkey* had a much lower GI than *banku*. Also, local and industry‐prepared *fufu* are both cassava and plantain based but have different GIs. The main explanation to these variations is processing.

Generally and for most foods, boiling is considered to increase GI due to increased gelatinization which improves starch digestibility and increased glucose response (Lin et al. 2010; Bahado‐Singh et al. 2011). This could explain the high GI obtained with *banku* which was low in fiber and had larger 50 g available carbohydrate portion. Also *banku* is prepared from corn dough and a small amount (20%) of cassava dough. The cassava could have influenced GI obtained. Cassava has a higher amylopectin to amylose ratio (United States Department of Agriculture, 2002). Amylopectin is more branched and more susceptible to digestive amylases and would thus increase glucose response (Arvidsson‐Lenner et al. 2004).

Of all the corn‐based foods (*kenkey, banku,* and *TZ*), however, *kenkey* had the least glycemic response (Table 2). The low GI value obtained for Ga *kenkey* was in agreement with a study done by Brakohiapa et al. (1997) on the glucose response to some mixed Ghanaian diet, in which it was reported that *kenkey* induced a low glucose response on consumption by healthy individuals. The low GI for *kenkey* may be due in part to high fiber content (2.46/100 g) and lower available carbohydrate portion per 100 g of test food compared to the other corn‐based foods tested (*TZ,* 0.3/100 g and *banku*, 0.1/100 g of fiber).

Fiber delays gastric emptying and this has an influence on glucose response by the body (Lin et al. 2010). Fiber is a nondigestible starch and together with some other nonstarch polysaccharides enters the large intestines to undergo fermentation into short‐chain fatty acid products like butyrate, propionate, and acetate. In the preparation of *kenkey*, the corn is allowed some days in water to ferment. Some organic acids such as acetic acid considered as a part of normal diet and formed during sourdough fermentation (Ostman et al. 2005) could have been produced during the fermentation of corn for *kenkey* preparation. The acid (which contributes to its characteristic taste and flavor) could have influenced the glycemic response of *kenkey*. There have been reports of the improvement in glycemic control to starch following fermentation of vegetables (Ostman et al. 2001) and other foods. Earlier studies had hinted the increased potential of weak acids with lower molecular weight (e.g., acetic acid, M_w_ = 60 g/mol) to delay gastric emptying and thus reduce glycemic response (Ostman et al. 2005). Liljeberg and Bjorck (1998) underscored the influence of acetic acid on glycemic response and even suggested the inclusion of fermented foods in meals to improve glycemic control (Liljeberg and Bjorck 1998). They further affirmed that acetic acids reduced glycemic response by delaying gastric emptying. The acids from the fermentation of corn in *kenkey* preparation could have decreased the glycemic response by same mechanism of delayed gastric emptying. These might explain the difference in GI between *kenkey* and *banku*.

*Kenkey* preparation process requires a partial cooking of the fermented dough, followed by cooling to mix with uncooked dough, molding, and then final boiling. In *TZ* preparation, cool corn powder is added as cooking continued. These heating and cooling cycles in *kenkey* preparation are lacking in *TZ* preparation and have the likelihood of leading to the formation of retrograded starches (Bahado‐Singh et al. 2011). Retrograded starches are recrystallization product of starches that have formed due to strong intermolecular hydrogen bonding and are thus less susceptible to enzymatic breakdown. Increased amounts of retrograded starch increase the value of resistant starches in the boiled food. This can reduce glucose response and lead to a lower GI and may explain the differences in GI observed between *kenkey* and *TZ*, which are both completely corn based.

The values obtained and differences observed in the GI values of the corn‐based foods are similar to what is observed in the Revised International Table of Glycemic Index and Glycemic load where corn granules consumed commonly in China had a GI of 52 ± 3, maize (*Zea mays*) flour made into chapatti in India had GI of 59 and corn meal porridge in China which is similar to local corn porridge or very soft *banku* reported a GI of 68 ± 3 (Atkinson et al. 2008).

In comparison between the corn‐based and cassava‐based staples, although *banku* and locally pounded *fufu* (LPF) had 0.1 g fiber per 100 g (Eyeson et al. 1975), their glycemic responses differed, *banku* having a higher GI than LPF (Table 1). The *banku* was prepared from corn and cassava dough while the LPF from plantain and cassava. The two foods could not be compared without bias on the basis of their fiber content alone because differences in the amylose–amylopectin ratios of their starch structure and different processing methods are important factors in the rate of starch digestion and important influence on their glycemic responses.

From Figure 2, there is also an observable difference between the glucose response as elicited by LPF and IPF. A number of factors could have influenced the observed differences in glycemic response. Though the major component in the preparation of both LPF and IPF is plantain, the quantity differed per 100 g of each. The LPF was 80% plantain and 20% cassava while the IPF was 60% plantain, 10% cassava, and 30% granular potatoes (which were added to protect flavor). The influence of fiber on the different glycemic responses cannot be fully examined and compared since this was not stated in the industrially processed *fufu*.

The different processing methods and the variation in composition of raw materials of both *fufu* types (IPF and LPF) could have significantly influenced the glycemic responses. The industrial processes involved in the production of *fufu* flour, and subsequent cooking of the flour to produce the finished *fufu* product, involved a number of wetting, heating ,and cooling cycles (blanching, hot air drying, and cooking) (Johnson et al. 2006). These temperature‐induced processes affect starch digestibility and influence glycemic response (Brand et al. 1985). Higher processing temperatures lead to a disruption of the starch structure and increase digestibility (Lin et al. 2010; Bahado‐Singh et al. 2011). However, the industrial processing of the *fufu* flour involves blanching (Johnson et al. 2006) which does not expose the raw materials (plantain, cassava, potato) to extreme temperatures to completely disrupt the starch structure. The drying temperature is also not beyond 60**°**C (Johnson et al. 2006). Conversely, the heating and drying cycles during the production of the flour and the cooking of the flour to *fufu* could have rather increased the amount of retrograded starch (R3‐resistant starch) present in the flour, which are less susceptible to enzymatic breakdown (Bahado‐Singh et al. 2011), and thus lower glycemic response. To add to that, the 30% granular potatoes in industrially processed *fufu* could have led to low GI of processed *fufu* flour. Studies by Elmståhl in 2002 revealed a high‐resistant starch content of processed potatoes. The contribution of R2‐ resistant starch (Elmstahl 2002) from the 30% processed potato fraction of the *fufu* flour could have significantly influenced the lowered glycemic response of the IPF as compared to locally pounded *fufu*.

In the study there were no restrictions on evening meals prior to testing days. As shown in Figure 1 and 2, there were not much difference in the fasting blood sugar levels before any of the test or reference foods were consumed though the subjects consumed different meals the evenings prior to testing days. The interindividual variations in glycemic responses are in agreement with studies by Brouns et al. (2005) (United States Department of Agriculture, 2002). Since the previous evening meals were not controlled, the interindividual variations in GI of foods could not be attributed wholly to previous evening meals though the possibility could not be ruled out. According to a study by Thorburn et al. (1993), low GI foods especially fermentable high fiber evening meals like barley improved glucose tolerance to a breakfast meal the following morning compared to rice. Wolever in 1988 studied the second meal effect on GI and concluded that high fiber in an evening meal without recourse to the GI of the meal necessarily, could influence the glucose response of the morning meal (Wolever et al. 1988). A similar finding was observed in a study by Granfeldt et al. (2005) on the effect of high fiber evening meal on GI.

This study could have been limited by a few factors. For example, because the test foods could not be taken raw, they were all taken with the same quantity and quality of light soup and about a 30 g size of salmon fish. This was to ensure that there were no differences that could be attributed to the soup taken with a particular food item. The soup used, added very little variation to the fiber and available carbohydrate portions that are important components to the GI measurements. The light soup together with the fish provided a total of 6.96 g of protein, 2.52 g of fat, and 2.31 g of available carbohydrate with no fiber.

Studies on protein hydrolysates in meals have showed significant effect in insulin and glucagon responses. The effect was, however, dependent on the type of protein at significant quantities given per body weight (Claessens et al. 2009). Earlier researches into co‐ingestion of carbohydrates with protein, however, showed increases in response of plasma insulin (Rabinowitz et al. 1966; Newsholme et al. 2005). The effect of proteins on the glucose raising ability of foods were not, however, elucidated in these studies, though insulin secretion would influence the AUC of glucose response curve. In a study conducted in type II diabetics where participants were given each 50 g glucose plus 25 g of different proteins including egg white, the highest glucose response was found in glucose ingestion alone or glucose ingested with egg white (Gannon et al. 2001). This informs the position that the egg white did not counter influence glucose response and that for a protein to influence glucose response, the quality, and quantity of the protein is critical as indicated by Newsholme et al. (2005) (Lavigne et al. 2000). The total amount of protein from the meat and soup in this study being 6.96 g was similar to the total protein content of *kenkey* but significantly lower than the total protein content of *banku*. With the same amount of soup and meat taken with all the foods and the responses observed, it was not likely there was any significant effect of the very low protein value on the IAUCs of the various foods tested. Furthermore, studies that demonstrated significant effect of protein on glucose response or even insulin response required significant quantities of defined proteins (Lavigne et al. 2000; Claessens et al. 2009).

The soup and fish provided a total of 2.51 g fat to the tested foods. This was the same throughout all the tested foods and could not have influenced the glucose response significantly. The amount of fat that could influence glycemic response should be enough to influence the physicochemical properties of the food as with frying demonstrated by Bahado‐Singh et al.*,* 2011 in studying the effect of various processing methods on sweet potatoes (*Ipomoea batatas*) (Bahado‐Singh et al. 2011). This was not likely with the amount of fat from the soup and fish because it was almost of the same value as that present in the various tested foods.

The salmon added had zero available carbohydrate portion. The light soup, however, had approximately 2 g available carbohydrate which could be considered within an acceptable standard deviation for the measured amount of available carbohydrate portion of the food tested and thus provide no significant increase in the glucose response of the tested foods.

## Conclusion

This study has established the GI of five major carbohydrate‐rich staples consumed in Ghana and some parts of Africa. A low to moderately high GIs were observed indicating that these staples are of different carbohydrate quality and will influence blood glucose differently. These should be considered in recommendations for diabetics. The results from this study also affirm the position that the GI of individual foods should be tested and not extrapolated from foods that have similar descriptions (Aston et al. 2008). Further work is recommended to determine the GI of all carbohydrate‐rich locally available Ghanaian foods, which are commonly consumed but whose GIs are currently unknown.

## Conflict of Interest

None declared.
